# Efficient ^1^H-NMR Quantitation and Investigation of *N*-Acetyl-d-glucosamine (GlcNAc) and *N*,*N′*-Diacetylchitobiose (GlcNAc)_2_ from Chitin

**DOI:** 10.3390/ijms12095828

**Published:** 2011-09-09

**Authors:** Fu-Chien Liu, Chung-Ren Su, Tzi-Yi Wu, Shyh-Gang Su, Huey-Lang Yang, John Han-You Lin, Tian-Shung Wu

**Affiliations:** 1 Department of Chemistry, National Cheng Kung University, Tainan 701, Taiwan; E-Mails: t718z@yahoo.com.tw (F.-C.L.); t0718z@gmail.com (C.-R.S.); t718z@hotmail.com (T.-Y.W.); z7902010@email.ncku.edu.tw (S.-G.S.); 2 Institute of Biotechnology, National Cheng Kung University, Tainan 701, Taiwan; E-Mails: hlyang@mail.ncku.edu.tw (H.-L.Y.); hanyou@mail.ncku.edu.tw (J.H.-Y.L.); 3 Department of Pharmacy, China Medical University, Taichung 401, Taiwan

**Keywords:** nuclear magnetic resonance, hydrolysis, kinetics, chitin, enzyme

## Abstract

A quantitative determination method of *N*-acetyl-*d*-glucosamine (GlcNAc) and *N*,*N′*-diacetylchitobiose (GlcNAc)_2_ is proposed using a proton nuclear magnetic resonance experiment. *N*-acetyl groups of GlcNAc and (GlcNAc)_2_ are chosen as target signals, and the deconvolution technique is used to determine the concentration of the corresponding compound. Compared to the HPLC method, ^1^H-NMR spectroscopy is simple and fast. The method can be used for the analysis of chitin hydrolyzed products with real-time analysis, and for quantifying the content of products using internal standards without calibration curves. This method can be used to quickly evaluate chitinase activity. The temperature dependence of ^1^H-NMR spectra (VT-NMR) is studied to monitor the chemical shift variation of acetyl peak. The acetyl groups of products are involved in intramolecular H-bonding with the OH group on anomeric sites. The rotation of the acetyl group is closely related to the intramolecular hydrogen bonding pattern, as suggested by the theoretical data (molecular modeling).

## 1. Introduction

*N*-acetyl-d-glucosamine (GlcNAc) and its derivative *N,N′*-diacetylchitobiose (GlcNAc)_2_ are a class of medicinal agent [1–5] with several interesting pharmacological activities, such as therapeutic activity in osteoarthritis [6] and inflammatory bowel disease [7]. They have also been evaluated as food supplements [8]. The hydrolysis of chitin may produce a variety of products, such as monomer GlcNAc, dimer (GlcAc)_2_, and chitosan oligomer. They have recently received considerable attention as functional materials for many applications in the fields of medicine, the food industry, agriculture, cosmetics and pharmaceuticals [9]. Although GlcNAc and (GlcNAc)_2_ can be produced via the acid hydrolysis of chitin, this procedure has high costs, low yields, and acidic waste [10]. Recently, GlcNAc and (GlcNAc)_2_ has been produced from chitins using enzymatic hydrolysis with good yields [11,12]. Therefore, methods that allow an accurate measurement of GlcNAc and (GlcNAc)_2_ are needed. Several methods have been used to determine the content of chitin hydrolyzed products, such as thin layer chromatography (TLC) [13] and high-performance liquid chromatography (HPLC) [12,14,15]. The most widely used method for analyzing the chitinase-catalyzed hydrolyzed products of chitin is HPLC. However, this method requires that enzymes be deactivated and filtered through a dialytic membrane before HPLC analysis. Calibration curves are also needed to quantitatively determine the content of chitin hydrolyzed products.

**Scheme 1:**
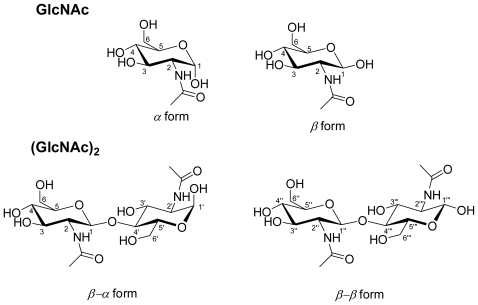
Structures of *N*-acetyl-d-glucosamine (GlcNAc) and *N*,*N′*-diacetylchitobiose (GlcNAc)_2_.

A highly specific and sensitive method is developed in this paper. Here, we describe the quantitative analysis of GlcNAc and (GlcNAc)_2_ simultaneously using an ^1^H-NMR experiment. Compared to the HPLC method, ^1^H-NMR spectroscopy quickly provides more information on chitin hydrolyzed products (*α*/*β* GlcNAc, *β*-*α*/*β*-*β* (GlcNAc)_2_, Scheme 1). No standard compounds are required for the preparation of calibration curves. All the components are detected simultaneously in a single measurement without any pre-cleaning steps. To our knowledge, no quantitative NMR method has been developed and validated for GlcNAc and (GlcNAc)_2_ determination. In the present study, an ^1^H-NMR method was developed for analyzing chitinase-catalyzed hydrolysis products obtained from chitin. This method can be used for the analysis of hydrolysis products obtained from chitin without destroying enzyme activity, and for quantifying the content of products using internal standards without calibration curves. This method has an excellent correlation between the concentration of products and the integration of the peak, and the ratio’s of α,β configurations of products. The experiments were carried out in phosphate buffer solutions with various pH levels to estimate the activity of chitinase. This study also focused on the kinetic behavior of *N*-acetyl-d-glucosamine and *N*,*N′*-diacetylchitobiose in pyridine-*d*_5_. The rate constants were associated with the variation of chemical shifts at the acetyl peak of GlcNac and (GlcNAc)_2_, respectively, and were studied using the temperature dependence of ^1^H-NMR spectra (VT-NMR). Density-functional theory (DFT) studies with Becke’s three-parameter hybrid function using the Lee, Yang, and Parr correlation function (B3LYP) were performed on the equilibrium geometries for *N*-acetyl-*d*-glucosamine and *N*,*N′*-diacetylchitobiose. The optimized geometric structure and the geometrical parameters (bond lengths and energy) of intramolecular hydrogen bonds in GlcNAc were also studied.

## 2. Results and Discussion

### 2.1. Quality Analysis of Enzyme Hydrolyzed Products

An ^1^H NMR method was developed to analyze the chitinase-catalyzed chitin hydrolysis products. This method can be used for the analysis of chitin hydrolysis products without destroying of the enzyme activity, and to quantify the contents of products using internal standard without need of the calibration curves. Especially, this method has an excellent correlation between concentration of the products and integration of the peak, also the ratio’s of α,β conformers of products.

**Figure 1:**
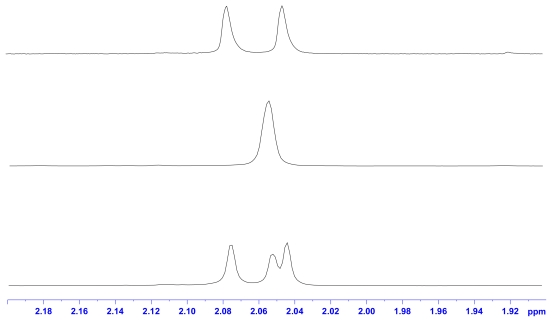
^1^H-NMR spectroscopy of *N*-acetyl group of GlcNAc, (GlcNAc)_2_, and the hydrolyzates of chitin by chitinase at 290 K.

**Figure 2:**
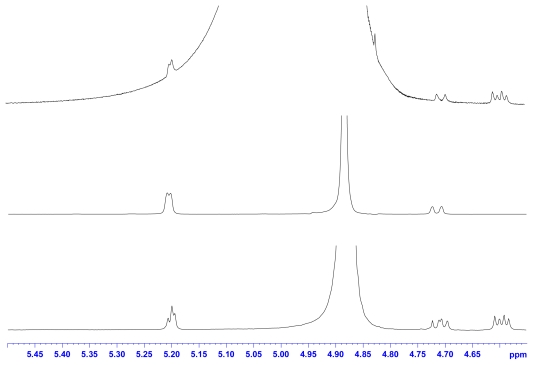
^1^H-NMR spectroscopy of anomeric protons of GlcNAc, (GlcNAc)_2_, and the hydrolyzates of chitin by chitinase at 290 K.

With the enzyme catalysis of chitin, two kinds of hydrolysate were obtained: *α*/*β* GlcNAc and *β*-*α*/*β*-*β* (GlcNAc)_2_ (Scheme 1). The analysis of the ^1^H-NMR spectrum of the standard GlcNAc, (GlcNAc)_2_, and hydrolysates shows that *N*-acetyl groups and anomeric protons can be selected as target signals for quantification (Figures 1 and 2). We chose TSP as an internal standard because it is a very stable compound with a simple ^1^H-NMR spectrum consisting of a singlet (*δ* 0.00). The quantities of the compounds were calculated using the relative ratio of the intensity of each compound to a known amount of the internal standard. The chemical shifts of *α*/*β* GlcNAc and *β*-*α*/*β*-*β* (GlcNAc)_2_ are quite easy to assign (Table 1), but some signals overlap. This can be solved using the deconvolution routine of Bruker NMR software, making the *N*-acetyl groups of GlcNAc and (GlcNAc)_2_ distinguishable. The deconvolution allows the determination of the individual areas and the line widths of the overlapped signals. Thus, it can be used for quantifying GlcNAc and (GlcNAc)_2_. In addition, anomeric protons of *α*/*β* GlcNAc and *β*-*α*/*β*-*β* (GlcNAc)_2_ appeared in the range 5.3–4.5 ppm; therefore, this is the working region for determining the ratio of *α*/*β* GlcNAc and the ratio of *β*-*α*/*β*-*β* (GlcNAc)_2_. However, when acquiring an NMR spectrum at 305 K, the signals H*_β_*-1 and H*_β_*-1″ of (GlcNAc)_2_ overlap with the H_2_O background signal. In order to prevent this overlap, the NMR working temperature was changed to 290 K. The signals H*_β_*-1and H*_β_*-1″ can then be used for quantifying the β-α and β-β forms of (GlcNAc)_2_ using the NMR deconvoluting technique. Furthermore, *α-*GlcNAc and *β-*GlcNAc can be determined by subtracting the overlapping signals from *α*/*β* GlcNAc and *β*-*α*/*β*-*β* (GlcNAc)_2_.

Calibration curves was determined in the concentration ranges for GlcNAc and (GlcNAc)_2_ to evaluate the accuracy of this method at various concentrations, respectively. The calibration curves were made using the ratio of the peak integral of the compound and the internal standard (TSP) (Table 2). The linearity of the calibration curves was determined by plotting the least squares regression lines. All calibration curves were highly linear with a *r*^2^-value of more than 0.999. Because all preliminary quantifications were well within the linear range of this method, we can conclude that these values were accurate. It should be noted that these calibration curves are not needed for quantifying the compounds in future experiments because the area of each signal is proportional to the molar concentration of the corresponding compound in ^1^H-NMR.

An HPLC method was used to confirm the analysis results obtained using the ^1^H-NMR method (Table 3). HPLC methods only obtain the total yield of GlcNAc and (GlcNAc)_2_; they cannot determine the ratio of *α*/*β* GlcNAc and the ratio of *β*-*α*/*β*-*β* (GlcNAc)_2_. Therefore, the NMR method is simple, rapid, and specific; no reference compounds are needed. An overall profile of the preparation can be obtained directly.

Using the proposed method, the production of GlcNAc and (GlcNAc)_2_ can be analyzed in a much shorter time than that required for conventional chromatographic methods. We report a systematic study on a commercial chitinase derived from *Streptomyces griseus* HUT-6037 (EC 3.2.1.14), which leads to a higher efficiency of GlcNAc and (GlcNAc)_2_ production from *α*-chitin. The experiments were carried out in different pH phosphate buffer solutions to estimate the activity of chitinase. This enzyme was tested for hydrolytic activity of *α*-chitin at pH 5~9 within 5 days and the amount of GlcNAc and (GlcNAc)_2_ by chitinase from *Streptomyces griseus* HUT-6037 at various pH values summaries in Table 4. The amount of (GlcNAc)_2_ is the highest at pH = 9, however, the amount of (GlcNAc) is the highest at pH = 5. When chitinase hydrolysis reaction was set in the acidic range, the yield of GlcNAc significantly increased with incubation time. However, the yield of (GlcNAc)_2_ reached its maximum after 3 days, and then decreased slowly. The results indicate that acid hydrolysis of (GlcNAc)_2_ might occur, resulting in GlcNAc production. When the pH was 7, GlcNAc and (GlcNAc)_2_ were produced at the same rate. After 5 days, the yield of GlcNAc was the same as that of (GlcNAc)_2_. A study on chitinolytic activity in the basic range showed that a significant yield of (GlcNAc)_2_ can be obtained observed at pH 9. However, the percent yield of GlcNAc production increased only slightly. The results indicate that the acid hydrolysis of (GlcNAc)_2_ does not happen and that it does not transfer to GlcNAc. The ratio of *α*/*β* GlcNAc and the ratio of *β*-*α*/*β*-*β* (GlcNAc)_2_ were also evaluated (Table 4). The yield of *β*-*α* (GlcNAc)_2_ was significantly higher than that of *β*-*β* (GlcNAc)_2_, while the yield of GlcNAc was higher for the *β* form.

### 2.2. Kinetic Properties of Enzyme Hydrolyzed Products

**Figure 3:**
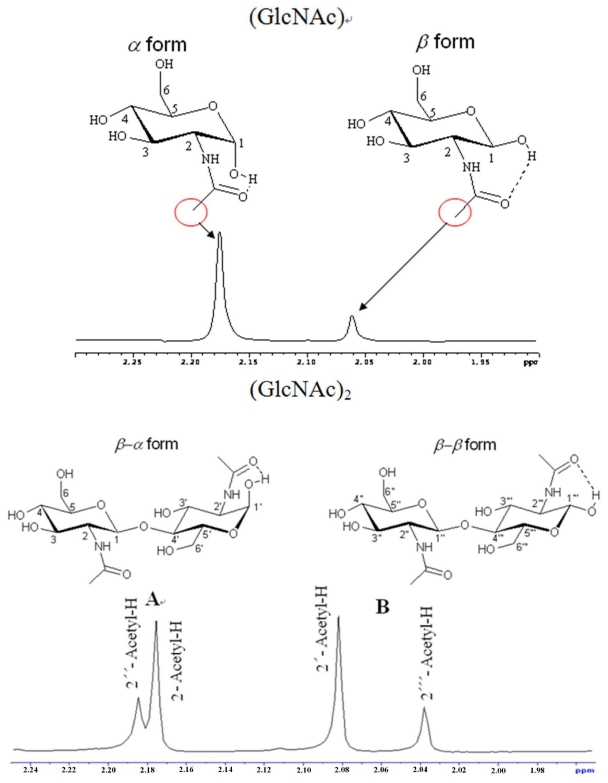
The ^1^H NMR signal of (GlcNAc) and (GlcNAc)_2_ in pyridine-*d_5_*.

The kinetic properties of enzyme hydrolyzed products were determined experimentally using VT-NMR. Figure 3 shows the ^1^H-NMR signal of (GlcNAc) and (GlcNAc)_2_ in pyridine-*d_5_*.

**Figure 4:**
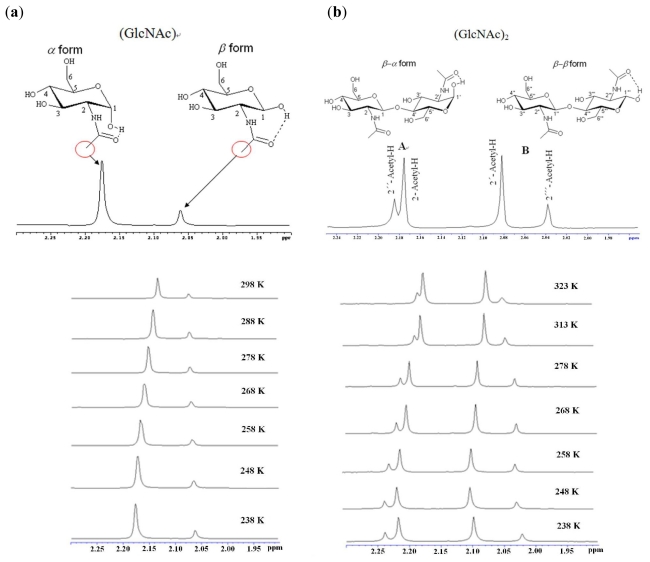
The chemical shift difference of (**a**) α/β form (GlcNAc) and (**b**) β-α/β-β form (GlcNAc)_2_.

We can evaluate the rate constant from Equation (1) at various temperatures [16].

(1) $$k={\left[{\left(\Delta v_{\text{o}}\right)}^{2}-{\left(\Delta v_{\text{p}}\right)}^{2}\right]}^{1/2}\times \pi /\sqrt{2}$$

where Δ*v*_o_ is the difference of chemical shifts of the acetyl peak (α form and β form) at the lowest temperature (*T* = 238 K). With the increase of temperature, the acetyl peak positions of GlcNAc α form and β form are close from 238 to 298 K (Figure 4). Δ*v*_p_ is the difference of chemical shifts of the acetyl peak at the present temperature (*T* = 248–343 K for (GlcNAc), and *T* = 248–338 K for (GlcNAc)_2_). The rate constant (*k*) of (GlcNAc) and (GlcNAc)_2_ at various temperatures is summarized in Table 5. The column headings “A” and “B” refer to the chemical shift difference of β-a/β-β form (GlcNAc)_2_. The temperature calibration of the VT-NMR has been studied using polynomial fitting (*k* = *a*_0_ + *a*_1_*T* + *a*_2_*T*^2^ + *a*_3_*T*^3^), the fitting parameters of kinetic constants (*k*) for (GlcNAc) and (GlcNAc)_2_ at various temperatures are summarized in Table 6.

From the ^1^H-NMR study of (GlcNAc)_2_ in pyridine-*d_5_*, the signal of the α-form acetyl group increases with reaction time; however, the signal of the β-form acetyl group decreases, implying opening and closing of the ring in (GlcNAc)_2_ [17–19]. The signal magnitude of the β-form acetyl group changes to the signal magnitude of the α-form acetyl group gradually, suggesting that the intramolecular hydrogen bonds of (GlcNAc)_2_ in pyridine-*d_5_* make α-form more stable than β-form.

### 2.3. Equilibrium Geometries

**Figure 5:**
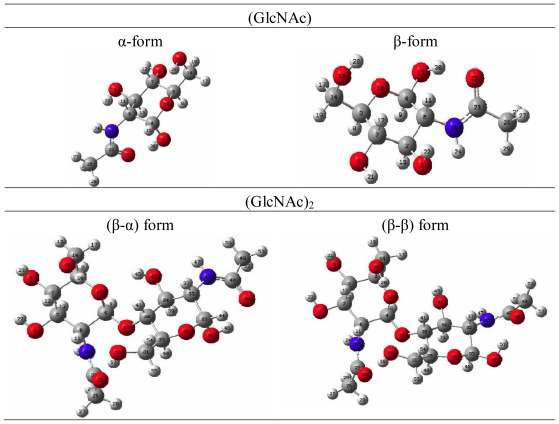
B3LYP optimized geometrical structure of (GlcNAc) and (GlcNAc)_2_.

Density functional theory (DFT) is a quantum mechanical method used in physics and chemistry to investigate the equilibrium geometric structure [20–26]. The geometrical properties of *N*-acetyl-d-glucosamine were studied with density functional theory (DFT) using the Gaussian 03 program package, as shown in Figure 5. The calculations were done on a B3LYP/6-31G(d) level for geometry optimizations in the ground state. B3LYP is a hybrid function modified from the three-parameter exchange-correlation functional of Becke [20], while the gradient-corrected exchange and correlation functions are calculated according to Becke [21] and Lee *et al*. [22]. The geometrical parameters (bond length, bond angle, and bond energy of intramolecular hydrogen bonds) were studied from the optimized equilibrium geometries. The geometric parameters of *N*-acetyl-d-glucosamine are summarized in Table 7. The bond lengths and bond angles of α-form *N*-acetyl-d-glucosamine are similar to those of β-form *N*-acetyl-d-glucosamine, except bond angle ∠O-H-O shows significant difference between α-form (∠O9-H30-O25 = 163.29°) and β-form (∠O15-H30-O25 = 150.35°) *N*-acetyl-d-glucosamine. The bond length and bond energy of intramolecular hydrogen bonds (O25-H30) from α-form *N*-acetyl-d-glucosamine are 1.78 Å and 10 kJ/mole, respectively, whereas those from β-form *N*-acetyl-d-glucosamine are 1.82 Å and 8 kJ/mole, respectively. There is a relationship of the conformational study of GlcNAc to the NMR experiments. α form GlcNAc shows shorter intramolecular hydrogen bond length 1.78 Å than β form GlcNAc (1.82 Å), the chemical shift of ^1^H NMR shows acetyl signal of β form GlcNAc is upfield than α form GlcNAc.

## 3. Experimental Section

### 3.1. Chemicals

*α*-chitin was purchased from Kiotek Corp (Hsinchu, Taiwan). Chitinase derived from *Streptomyces griseus* HUT-6037 (EC 3.2.1.14), and the reference compounds *N*-acetyl-d-glucosamine (GlcNAc) and *N*,*N′*-diacetylchitobiose (GlcNAc)_2_ were purchased from Sigma. D_2_O (99.9%) and TSP (3-(trimethylsilyl)-propionic-2,2,3,3-*d*_4_ acid sodium salts) were obtained from Aldrich. HPLC grade CH_3_CN was purchased from Merck (Darmstadt, Germany).

### 3.2. General

^1^H-NMR spectra were recorded in a deuterated phosphate buffer solution using a Bruker AVANCE 500 spectrometer at 290 K. For each sample, 32 scans were recorded with the following parameters: 0.19 Hz/point; spectra width, 5482.456 Hz; pulse width, 8 μs; relaxation delay, 2 s; and acquiring time, 2.988 s. For quantitative analysis, the peak area was used; the start and end points of the integration of each peak were selected manually. The relative area under the overlapping peak was analyzed using the Gaussian/Lorentzian deconvolution technique [27,28].

The ^1^H-NMR chemical shifts of *N*-acetyl-d-glucosamine (GlcNAc) and *N*,*N*′-diacetylchitobiose (GlcNAc)_2_ summarize as follows,

Acetyl signal:

*N*-acetyl-d-glucosamine (GlcNAc): δ 2.05(s).

*N*,*N*′-diacetylchitobiose (GlcNAc)_2_: δ 2.08(s), δ 2.05(s).

Anomeric signal:

(GlcNAc): H*_α_*-1′ signal: δ 5.21 (d, *J* = 3.0 Hz)

H_â_-1 signal: δ 4.72 (d, *J* = 8.4 Hz)

(GlcNAc)_2_: H_á_-1′ signal: *δ* 5.20 (d, *J* = 2.6 Hz)

H_â_-1‴ signal: δ 4.70 (d, *J* = 7.9 Hz)

H_â_-1 signal: δ 4.60 (d, *J* = 8.5 Hz)

H_â_-1″ signal: δ 4.59 (d, *J* = 8.5 Hz)

The internal standard of ^1^H-NMR spectrum is TSP (3-(Trimethylsilyl)propionic-2,2,3,3-*d_4_* acid sodium salt). After evaluating the integral area ratio of internal standard and chitin hydrolysis products, the amount of chitin hydrolysis products can be evaluated from internal standard, the values of chitin hydrolyzed products (GlcNAc) and (GlcNAc)_2_ were fitted using the equations:

(2) $$(\text{GlcNAc})(\text{M})=\frac{A\times Ms}{3\times V}$$

(3) $${\left(\text{GlcNAc}\right)}_{2}(\text{M})=\frac{A\times Ms}{6\times V}$$

where *A* is the integral area ratio, *Ms* is the mole amount of internal standard, *V* is the volume of solution (L).

The calibration curves of ^1^H-NMR spectrum were made using the ratio of the peak integral of the compound and the internal standard (TSP). The linearity of the calibration curves was determined by plotting the least squares regression lines. The calibration curves were highly linear with a *r*^2^-value of 0.9999 and 0.9994 for GlcNAc and (GlcNAc)_2_, respectively.

HPLC analysis of chitin hydrolyzed products was performed on a Shimadzu LC-10AT*_vp_* (Japan) (Cosmosil Sugar-D column (4.6 × 250 mm, 5 μm); CH_3_CN/H_2_O = 8/2; flow rate = 1.0 mL/min; injection, 20 μL; detection, UV at 210 nm). Calibration curves were determined in the concentration ranges for GlcNAc and (GlcNAc)_2_ to evaluate the accuracy of this method at various concentrations (see supplementary materials). The linearity of the calibration curves was determined by plotting the least squares regression lines. The calibration curves were highly linear with a *r*^2^-value of 0.9995 and 0.9999 for GlcNAc and (GlcNAc)_2_, respectively.

### 3.3. NMR Analysis of Products from Chitin Hydrolysis

*α*-chitin (5 mg) was suspended in 400 μL of deuterated phosphate buffer solution (pH 5~9) in a 5 mm NMR tube containing 50 μL 0.01 M TSP (internal standard). *Streptomyces griseus* HUT-6037 (1.4 mg) dissolved in deuterated phosphate buffer solution (100 μL, pH 5~9) was then added into the above solution and shaken at 37 °C. After the prescribed time, the signal of enzyme is really weak, the amounts of GlcNAc and (GlcNAc)_2_ were directly estimated using NMR. The quantification of chitinase hydrolyzed products continued to be recorded for 5 days of incubation. The deuterated phosphate buffer solution was prepared by 0.1 M citric acid buffer (pH = 5–6) and 0.1 M Tris buffer (pH = 7–9). For pH 5.0, the deuterated phosphate buffer solution was prepared using approximately a 50%: 50% mixture of 0.1 M citric acid to 0.1 M disodium hydrogen phosphate. For pH 6.0, the deuterated phosphate buffer solution was prepared using titrating pH of phosphate with citric acid. For pH = 7–9, 0.1 M Tris buffer is titrated to desired pH with HCl.

### 3.4. HPLC Analysis of Products from Chitin Hydrolysis

*α*-chitin (5 mg) was suspended in 400 μL of deuterated phosphate buffer solution (pH 7) in a 5 mm NMR tube. *Streptomyces griseus* HUT-6037 (1.4 mg) dissolved in deuterated phosphate buffer solution (100 μL, pH 7) was then added into the above solution and shaken at 37 °C. After the prescribed time, the reaction mixture was taken out, filtered, and analyzed using HPLC. The amounts of GlcNAc and (GlcNAc)_2_ in the reaction mixture were estimated from the calibration curve of the standard GlcNAc and (GlcNAc)_2_.

### 3.5. Computational Methods

Equilibrium geometries were obtained using the Gaussian 03 program package [29] and optimized using B3LYP (Becke’s three-parameter hybrid function with the Lee-Yang-Perdew correlation function). Under the optimized geometries, total energies were obtained at the Pople 6–31 + g(d) level.

## 4. Conclusions

A highly specific and sensitive method using ^1^H-NMR was developed for the quantitative determination of *α*/*β* GlcNAc and *β*-*α*/*β*-*β* GlcNAc. This technique has a good agreement with the HPLC method. Since enzymatic hydrolysis is conducted under quite mild conditions and high selectivity is achieved in the production of GlcNAc and (GlcNAc)_2_, the proposed method is useful for quickly evaluating chitinase activity and for finding a suitable chitinase which could produce high yields of GlcNAc and (GlcNAc)_2_. Especially, this method has an excellent correlation between concentration of the products and integration of the peak, also the ratio of α,β conformers of products. A systematic study on a commercial chitinase derived from *Streptomyces griseus* HUT-6037 (EC 3.2.1.14) is reported, which leads to a higher efficiency of GlcNAc and (GlcNAc)_2_ production from *α*-chitin. The experiments were carried out in phosphate buffer solutions (pH = 5–9) to estimate the activity of chitinase. The kinetic properties of enzyme hydrolyzed products were determined experimentally using VT-NMR. The rate constant (*k*) of (GlcNAc) and (GlcNAc)_2_ at various temperatures is evaluated. The geometrical parameters (bond length, bond angle, and bond energy of intramolecular hydrogen bonds) of *N*-acetyl-d-glucosamine were studied from the optimized equilibrium geometries. From the ^1^H-NMR study of (GlcNAc)_2_ in pyridine-*d_5_*, a mechanism of the *N*,*N′*-diacetylchitobiose open-close ring system in pyridine-*d*_5_ is proposed.

## Supplementary Information



## Figures and Tables

**Table 1:** The target ^1^H-NMR chemical shifts of GlcNAc and (GlcNAc)_2_.

Substance	^1^H shift (δ)	Assignment
	2.05 (s)	*N*-acetyl group
*α*/*β* GlcNAc	5.21 (d, *J* = 3.0 Hz)	H*_α_*-1
	4.72 (d, *J* = 8.4 Hz)	H*_β_*-1

	2.08 (s), 2.05 (s)	*N*-acetyl group
	5.20 (d, *J* = 2.6 Hz)	H*_α_*-1′
*β*-*α*/*β*-*β* [GlcNAc]_2_	4.70 (d, *J* = 7.9 Hz)	H*_β_*-1‴
	4.60 (d, *J* = 8.5 Hz)	H*_β_*-1
	4.59 (d, *J* = 8.5 Hz)	H*_β_*-1″

* Solvent: deuterated phosphate buffer, pH = 5.0, the concentration is 50%: 50% mixture of 0.1 M citric acid and 0.1 M disodium hydrogen phosphate.

**Table 2:** The ^1^H-NMR peak integral of the (GlcNAc)_2_ and the internal standard (TSP). Calibration curves was determined in the concentration ranges for (GlcNAc)_2_ to evaluate the accuracy of ^1^H-NMR at various concentrations.

No.	Area of δ 0 (TSP)	Area of δ 2.08 and 2.05 (GlcNAc)_2_		Real Concentration of (GlcNAc)_2_ (mM)	Calculate Concentration of (GlcNAc)_2_ (mM)
1	120.491	4.968	4.819	0.00909	0.11076
2	103.17	10.524	10.65	0.227	0.27987
3	110.806	20.455	20.791	0.4545	0.50759
4	100.272	27.589	27.258	0.681	0.75850
5	115.023	40.753	41.764	0.909	0.99705

* Solvent: deuterated phosphate buffer, pH = 5.0.

**Table 3:** Comparison of the quantitative determination of GlcNAc and (GlcNAc)_2._

HPLC			NMR		

Digestion Time (day)	(GlcNAc)_2_ (mM)	(GlcNAc) (mM)	Digestion Time (day)	(GlcNAc)_2_ (mM)	(GlcNAc) (mM)
1	3.782	1.575	1	3.708	1.624
2	4.435	2.453	2	4.309	2.551
3	4.581	2.900	3	4.542	2.973
4	4.921	3.919	3	4.978	3.941
5	5.067	4.834	4	5.064	4.866

* D_2_O phosphate buffer, pH = 7.0.

**Table 4:** Production of GlcNAc and (GlcNAc)_2_ by chitinase from *Streptomyces griseus* HUT-6037.

Digestion Time (day)	(GlcNAc)_2_ (mM)	*β*-*α* (%)	*β*-*β* (%)	GlcNAc (mM)	*α* (%)	*β* (%)
pH = 5						
1	3.554	55.5	44.5	2.616	58.7	41.3
2	4.529	55.7	44.3	3.907	58.6	41.4
3	4.716	55.9	44.1	5.620	58.8	41.2
4	4.521	54.9	45.1	6.013	58.4	41.6
5	4.191	55.3	44.7	7.036	58.0	42.0
pH = 6						
1	3.797	61.5	38.5	2.155	57.5	42.5
2	4.361	59.9	40.1	3.144	58.4	41.6
3	4.819	59.9	40.1	4.241	59.2	40.8
4	4.502	61.7	38.3	5.405	60.1	39.9
5	4.373	60.5	39.5	6.542	59.9	40.1
pH = 7						
1	3.708	59.9	40.1	1.624	58.9	41.1
2	4.309	60.2	39.8	2.511	58.4	41.6
3	4.542	61.5	38.5	2.973	56.7	43.3
4	4.978	60.2	39.8	3.941	59.3	40.7
5	5.064	58.1	41.9	4.866	61.3	38.8
pH = 8						
1	3.716	60.3	39.7	1.045	58.5	41.5
2	4.116	59.9	40.1	1.399	59.1	40.9
3	4.795	60.5	39.5	1.749	58.8	41.2
4	4.879	60.8	39.2	2.088	59.7	40.3
5	5.218	60.7	39.3	2.160	58.8	41.2
pH = 9						
1	3.859	55.6	44.4	1.039	60.5	39.5
2	4.347	56.0	44.0	1.476	58.3	41.7
3	4.609	54.2	45.8	1.611	61.6	38.4
4	4.890	55.4	44.6	1.725	59.7	40.3
5	5.346	55.9	44.1	2.087	60.8	39.2

Solvent: deuterated phosphate buffer, pH 5–9, which was prepared by 0.1 M citric acid buffer (pH = 5–6) and 0.1 M Tris buffer (pH = 7–9).

**Table 5:** The rate constant (*k*) of (GlcNAc) and (GlcNAc)_2_ at various temperatures.

GlcNAc		(GlcNAc)_2_			

Temperature (K)	*k* (s^−1^)	A		B	

		Temperature (K)	*k* (s^−1^)	Temperature (K)	*k* (s^−1^)
248	42.493	248	9.966	248	24.24
258	62.679	258	13.746	258	36.812
268	78.666	268	15.899	268	47.58
278	90.64	278	17.643	278	54.627
288	101.33	313	21.425	313	77.374
298	107.583	323	21.629	323	80.458
313	116.661	333	21.994	333	83.397
323	120.419	336	22.166	336	83.593
333	123.459	337	22.328	337	83.951
343	125.503	338	22.473	338	84.114

Solvent: pyridine-*d_5_*.

**Table 6:** Fitting parameters of kinetic constants (*k*) for (GlcNAc) and (GlcNAc)_2_ at various temperatures (*k* = *a*_0_ + *a*_1_*T* + *a*_2_*T*^2^ + *a*_3_*T*^3^).

	a_0_	a_1_	a_2_	a_3_	R^2a^
GlcNAc	−2.898 × 10^3^	26.15	−7.612 × 10^−2^	7.461 × 10^−5^	0.9998
(GlcNAc)_2_ peak A	−6.184 × 10^2^	5.82	−1.776 × 10^−2^	1.82 × 10^−5^	0.9984
(GlcNAc)_2_ peak B	−9.782 × 10^2^	7.653	−1.789 × 10^−2^	1.345 × 10^−5^	0.9995

a Correlation coefficient.

**Table 7:** The bond length and bond angle, bond energy of intramolecular hydrogen bond of α and β form (GlcNAc) performed with the Gaussian 03 program.

		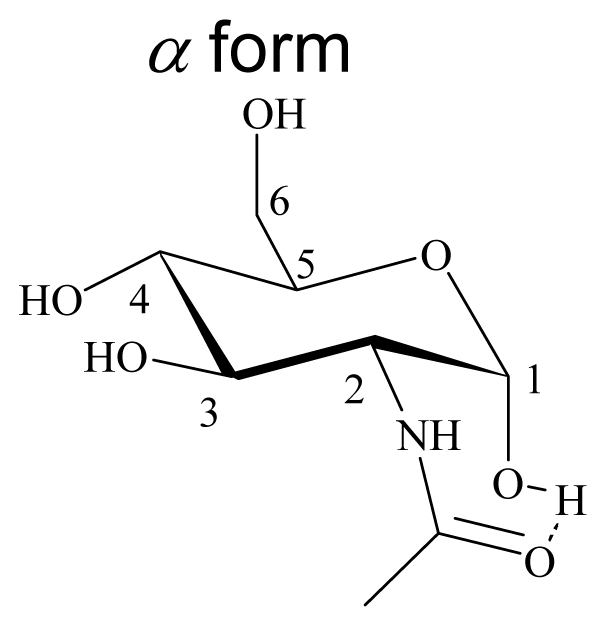	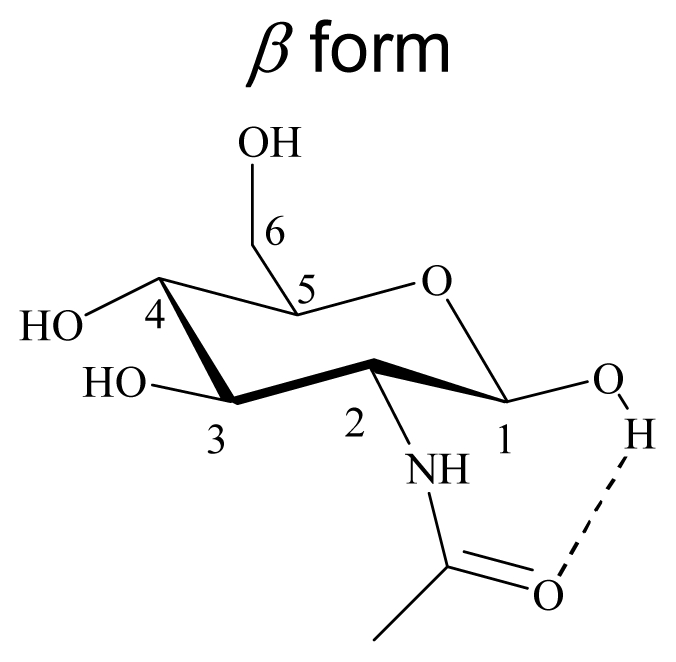
	C-O	1.39 Å (C4-O9)	1.37 Å (C4-O15)
	O-H	0.98 Å (O9-H30)	0.98 Å (O15-H30)
	C=O	1.24 Å (C23-O25)	1.23 Å (C23-O25)
Bond length	C-N	1.36 Å (C23-N16)	1.36 Å (C23-N16)
	C-C	1.52 Å (C23-C26)	1.52 Å (C23-C26)
	N-C	1.47 Å (N16-C6)	1.46 Å (N16-C6)
	C-C	1.55 Å (C6-C4)	1.55 Å (C6-C4)

	∠C-O-H	109.96° (∠C4-O9-H30)	109.36° (∠C4-O15-H30)
	∠O-H-O	163.29° (∠O9-H30-O25)	150.35° (∠O15-H30-O25)
	∠H-O-C	107.78° (∠H30-O25-C23)	107.78° (∠H30-O25-C23)
Bond angle	∠O-C-N	122.91° (∠O25-C23-N16)	122.91° (∠O25-C23-N16)
	∠C-N-C	126.17° (∠C23-N16-C6)	126.17° (∠C23-N16-C6)
	∠N-C-C	114.89° (∠N16-C6-C4)	114.89° (∠N16-C6-C4)
	∠O-C-C	114.19° (∠O9-C4-C6)	114.19° (∠O15-C4-C6)

Intramolecular hydrogen bond (Bond length)	O-H	1.78 Å (O25-H30)	1.82 Å (O25-H30)

Intramolecular hydrogen bond (Bond energy)	O-H	10 kJ/mole (O25-H30)	8 kJ/mole (O25-H30)
