# Supplementary material for: Efficient 1H-NMR Quantitation and Investigation of N-Acetyl-d-glucosamine (GlcNAc) and N,N′-Diacetylchitobiose (GlcNAc)2 from Chitin
Source: Int J Mol Sci. 2011 Sep 9;12(9):5828–43. doi: 10.3390/ijms12095828 (PMC3189753; doi:10.3390/ijms12095828)
Supplement: Supplementary file 1 [file ijms-12-05828-s001.pdf]

Supplementary Information

Fu-Chien Liu [1], Chung-Ren Su [1], Tzi-Yi Wu [1], Shyh-Gang Su [1], Huey-Lang Yang [2], John Han-You Lin [2] and Tian-Shung Wu [1,3,*]

[1] Department of Chemistry, National Cheng Kung University, Tainan 701, Taiwan; E-Mails: t718z@yahoo.com.tw (F.-C.L.); t0718z@gmail.com (C.-R.S.); t718z@hotmail.com (T.-Y.W.); z7902010@email.ncku.edu.tw (S.-G.S.)

[2] Institute of Biotechnology, National Cheng Kung University, Tainan 701, Taiwan; E-Mails: hlyang@mail.ncku.edu.tw (H.-L.Y.); hanyou@mail.ncku.edu.tw (J.H.-Y.L.)

[3] Department of Pharmacy, China Medical University, Taichung 401, Taiwan

* Author to whom correspondence should be addressed; E-Mail: tswu@mail.ncku.edu.tw; Tel.: +886-6-2757575; Fax: +886-6-2740552.

Received: 20 June 2011; in revised form: 22 August 2011 / Accepted: 26 August 2011 / Published: 9 September 2011

Abstract: A quantitative determination method of *N*-acetyl-*D*-glucosamine (GlcNAc) and *N,N'*-diacetylchitobiose $(GlcNAc)_2$ is proposed using a proton nuclear magnetic resonance experiment. *N*-acetyl groups of GlcNAc and $(GlcNAc)_2$ are chosen as target signals, and the deconvolution technique is used to determine the concentration of the corresponding compound. Compared to the HPLC method, ^{1}H-NMR spectroscopy is simple and fast. The method can be used for the analysis of chitin hydrolyzed products with real-time analysis, and for quantifying the content of products using internal standards without calibration curves. This method can be used to quickly evaluate chitinase activity. The temperature dependence of ^{1}H-NMR spectra (VT-NMR) is studied to monitor the chemical shift variation of acetyl peak. The acetyl groups of products are involved in intramolecular H-bonding with the OH group on anomeric sites. The rotation of the acetyl group is closely related to the intramolecular hydrogen bonding pattern, as suggested by the theoretical data (molecular modeling).

Keywords: nuclear magnetic resonance; hydrolysis; kinetics; chitin; enzyme

---

HPLC analysis of chitin hydrolyzed products was performed on a Shimadzu LC-10AT$_{vp}$ (Japan) (Cosmosil Sugar-D column (4.6 × 250 mm, 5 μm); CH_3CN/H_2O = 8/2; flow rate = 1.0 mL/min; injection, 20 μL; detection, UV at 210 nm).

Figure S1. HPLC chromatograms of background.

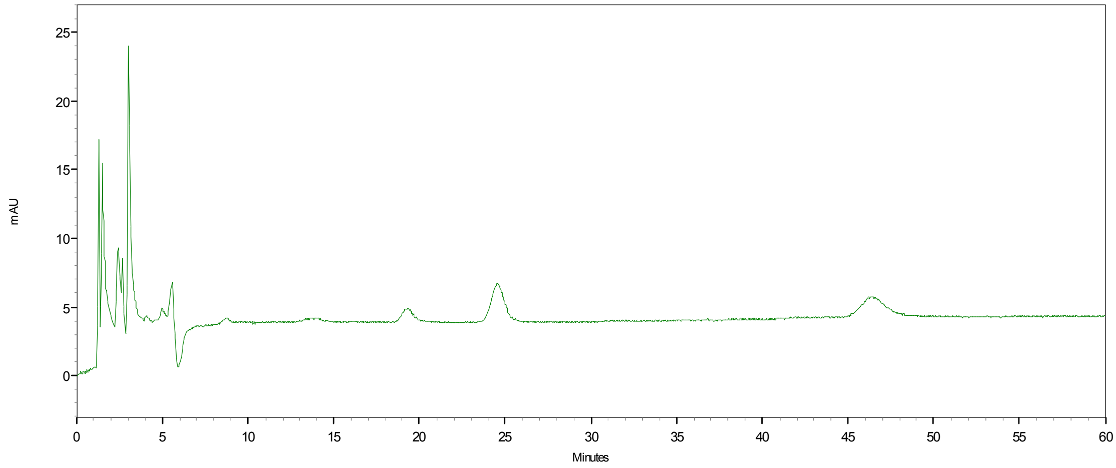

Figure S2. HPLC chromatograms of products from chitin hydrolysis.

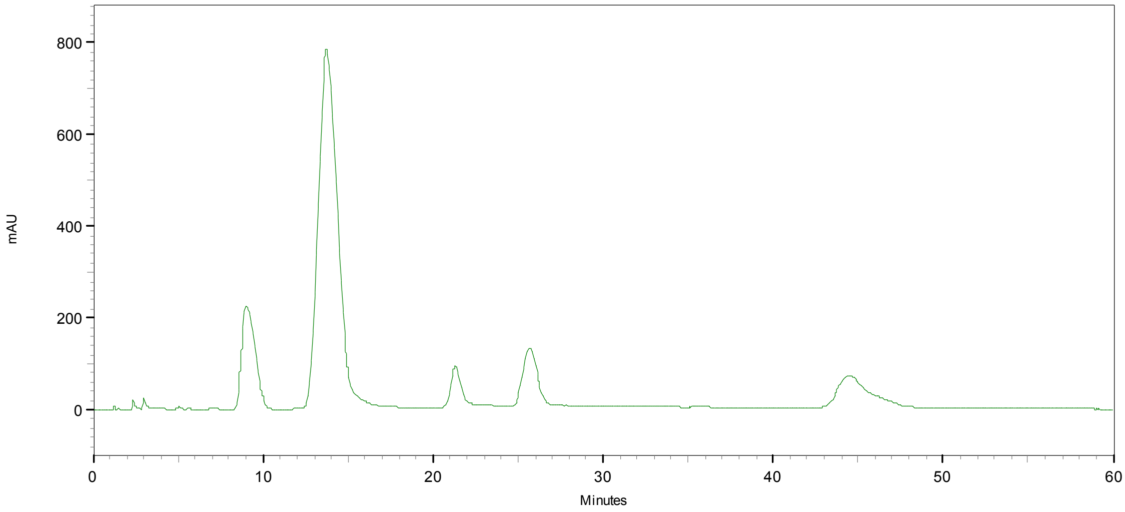

The retention times of GlcNAc and $(GlcNAc)_2$ are 9.0 and 13.7 min, respectively.

© 2011 by the authors; licensee MDPI, Basel, Switzerland. This article is an open access article distributed under the terms and conditions of the Creative Commons Attribution license (http://creativecommons.org/licenses/by/3.0/).
